# Prevalence and changes in chronic diseases among South Korean farmers: 1998 to 2005

**DOI:** 10.1186/1471-2458-9-268

**Published:** 2009-07-29

**Authors:** Eun Shil Cha, Kyoung Ae Kong, Eun Kyeong Moon, Won Jin Lee

**Affiliations:** 1 Department of Preventive Medicine, College of Medicine, Graduate School of Public Health, Korea University, Seoul, Korea

## Abstract

**Background:**

Epidemiologic studies have suggested a unique pattern of disease among farmers in Western countries, but limited information is available about the magnitude of disease prevalence and their changes over time in Asian farmers. The aim of this study was to compare the prevalence and changes in chronic diseases among farmers with those of other occupational groups in South Korea.

**Methods:**

Using data from three consecutive cross-sectional national surveys: the Korean National Health and Nutrition Examination Survey 1998 (n = 39,060), 2001 (n = 37,769), and 2005 (n = 34,145), we calculated age and gender-standardized prevalence of chronic diseases by the direct method and compared the prevalence changes from 1998 to 2005.

**Results:**

Female farmers had significantly higher chronic disease prevalence than other occupational groups in all three surveys. Arthritis was the most prevalent chronic disease among farmers for both men and women. Compared with other populations, farmers demonstrated a higher prevalence of arthritis and intervertebral disc disorders. Farmers showed higher prevalence changes for intervertebral disc disorders than other occupational workers.

**Conclusion:**

Our findings support that South Korean farmers have a distinct pattern of diseases prevalence from other populations. More detailed studies investigating the risk of musculoskeletal diseases and intensive intervention efforts to reduce the prevalence these diseases, particularly among female farmers, are required.

## Background

With over one billion workers, agriculture accounted for nearly 35% of worldwide employment [1]. A number of studies have suggested a unique pattern of morbidity and mortality among farmers [2-4]. Farmers have a remarkable deficit in total mortality, cardiovascular diseases, diabetes mellitus, psychiatric disorders and total cancer compared with the other populations, but appear to have higher prevalence rates of musculoskeletal diseases, respiratory diseases, hearing loss, skin disorders, accidental death and cancer of the skin, stomach, brain, and prostate, as well as the nervous, lymphohematopoietic systems [2,3,5,6].

Farming as an occupation has a number of unique characteristics compared to other occupations, such as worker traits and behavior, the work setting, and organizational structure [7]. Farming is not organized to provide workers with safety regulations or stable and flexible finances [8]. Farmers are further exposed to a wide range of occupational hazards such as ergonomic stresses, sunlight, viruses, inorganic dust, and pesticides [9].

Most previous investigations on farmers, however, have been conducted in the United States and Western Europe; so much less is known about the health status of farmers in Asian populations. Environmental and lifestyle factors for disease prevalence among farmers are likely to vary between countries. Although South Korea has traditionally been an agricultural country and retains a farm population exceeding 3.2 million people [10], few epidemiologic studies have focused on the farm sector. Most available epidemiologic studies on farmers in South Korea were focused on specific regions or symptoms, and did not estimate disease prevalence among the entire farm population. In addition, interpretation of these findings is limited due to the lack of an appropriate reference population [11].

This study, therefore, aimed to investigate chronic disease prevalence among farmers compared to other populations and relevant changes from 1998 to 2005 in order to identify major health issues among farmers in South Korea.

## Methods

### Measurement

The data used in this study was drawn from the 1998, 2001, and 2005 Korean National Health and Nutrition Examination Surveys (KNHNES). The KNHNES was a cross-sectional and nationally representative survey conducted by the Korean Ministry of Health and Welfare. The objective of the KNHNES is to estimate representative nationwide data about health status, utilization of health care and health-related behaviors and to provide a base for an effective health promotion program. In this survey, trained interviewers visited subjects' homes and administered a standardized questionnaire on the demographics, socioeconomic status, utilization of health care resources and medical history of each respondent. The target population of this survey included all non-institutionalized civilian South Korean individuals. The survey employed a stratified multistage probability sampling design, and the subjects were selected from sampling units predicated by geographical area, gender and age, as determined by household registries. The overall response rates of the Korean National Health and Nutrition Examination Survey were 90.8% in 1998, 92.3% in 2001, and 89.9% in 2005, respectively [12].

Participants were considered to have a chronic disease if they answered "yes" to both of these interview questions: "Have you experienced any chronic diseases in the preceding 12 months?" and "Have you been diagnosed with the disease by a physician?" Chronic diseases were defined as diseases lasting at least three months. Overall prevalence of chronic diseases in this study was defined by having any chronic disease except dental diseases. Individual chronic diseases were selected based on *a priori *interest in farmer's health and in having a sufficient number of cases for all three surveys. They included arthritis, intervertebral disc disorders, hypertension, chronic lung diseases, asthma, cancer, diabetes mellitus, and cataract/glaucoma.

### Study population

The total numbers of KNHNES participants were 39,060 (19,038 men and 20,022 women) in 1998, 37,769 (18,442 men and 19,327 women) in 2001, and 34,145 (16,356 men and 17,789 women) in 2005, respectively. We divided the total participants into three occupational groups of self-reported farmers, manual workers, and non-manual workers, based on the Korea standard classification of occupation [13]. The category of farmers including about 4.4% fishery and 0.2% forestry workers [10]. Manual workers included craft and related trades workers, plant/machine operators and assemblers, and elementary occupations, while non-manual workers included legislators, senior officials and managers, professionals, technicians and associate professionals, clerks, service workers and sale workers. We restricted the subjects to those aged 20 or more to examine prevalent chronic diseases and for comparison with working-age population. Through this, we investigated a 1998 population of 27,201 including 3,639 farmers, 4,677 manual and 8,101 non-manual workers, a 2001 population of 26,785 including 1,714 farmers, 4,996 manual and 9,193 non-manual workers, and a 2005 population of 25,161 including 1,732 farmers, 4,966 manual and 8,358 non-manual workers in the analysis.

### Statistical analysis

For all surveys, sampling weights were applied to account for unequal probabilities of selection resulting from the sample design, and from nonresponse, and to adjust for post-stratification population totals. Standard errors were calculated using Taylor series linearization method to account for the sampling design and to apply survey weight. Statistical analyses were carried out using Stata 10.0 (StataCorp., College Station, Texas). For comparisons across surveys, the age-standardized prevalence with 95% confidence intervals of chronic diseases was calculated using the direct adjustment method by 2005 standard population, with a population breakdown by five-year increments from the Korea National Statistical Office [10]. This analysis was performed separately for each of the three surveys and for gender. Differences in prevalence over time according to occupational groups were based on comparison of data from 2005 with 1998 data. This study analyzed publicly available data sets and was therefore exempt from institutional review board approval.

## Results

Table 1 illustrates the selected characteristics of the study subjects by occupational groups and the three surveys. Farmers were more likely than other occupational groups and the total population to be older, married, residing in rural areas, less educated, and collecting a lower income. Farmers and non-manual workers included a higher number of females than manual workers. Farmers were less often current smokers and drinkers of alcohol than other populations. Farmers had less frequent medical check-ups than other groups in 1998 and 2001, but not in 2005.

**Table 1:** Characteristics of participants aged 20 and older by occupational groups in three Korean National Health and Nutrition Examination Surveys

	1998				2001				2005			
	Farmers	Manual	Non-manual	Total^a^	Farmers	Manual	Non-manual	Total	Farmers	Manual	Non-manual	Total
	(n = 3,639)	(n = 4,677)	(n = 8,101)	(n = 27,201)	(n = 1,714)	(n = 4,996)	(n = 9,193)	(n = 26,785)	(n = 1,732)	(n = 4,966)	(n = 8,358)	(n = 25,161)
***Sex***												
Men	1,844 (50.7)	3,364 (71.9)	4,649 (57.4)	12,853 (47.3)	894 (52.2)	3,642 (72.9)	5,149 (56.0)	12,615 (47.1)	905 (52.3)	3,492 (70.3)	4,478 (53.6)	11,722 (46.6)
Women	1,795 (49.3)	1,313 (28.1)	3,452 (42.6)	14,348 (52.8)	820 (47.8)	1,354 (27.1)	4,044 (44.0)	14,170 (52.9)	827 (47.8)	1,474 (29.7)	3,880 (46.4)	13,439 (53.4)
***Age (years)***	55.3 ± 12.2	41.9 ± 11.3	37.7 ± 10.6	44.0 ± 15.7	57.6 ± 11.4	43.1 ± 11.1	38.4 ± 10.8	44.0 ± 15.5	59.4 ± 11.0	45.2 ± 11.5	39.1 ± 11.0	45.7 ± 15.8
***Educational level***												
≤ Elementary	2,450 (67.3)	1,191 (25.5)	623 (7.7)	7,729 (28.4)	1,151 (67.2)	994 (19.9)	544 (5.9)	5,837 (21.8)	1,103 (63.7)	997 (20.1)	420 (5.0)	5,561 (22.1)
Middle	577 (15.9)	1,056 (22.6)	742 (9.2)	3,705 (13.6)	293 (17.1)	1,031 (20.7)	700 (7.6)	3,285 (12.3)	300 (17.3)	924 (18.6)	514 (6.2)	2,873 (11.4)
High	544 (15.0)	2,156 (46.1)	3,287 (40.6)	9,572 (35.2)	227 (13.3)	2,456 (49.2)	3,540 (38.6)	9,820 (36.7)	274 (15.8)	2,479 (50.0)	2,833 (33.9)	8,618 (34.3)
≥ University	68 (1.9)	274 (5.9)	3,449 (42.6)	6,195 (22.8)	42 (2.5)	507 (10.2)	4,387 (47.8)	7,790 (29.1)	55 (3.2)	562 (11.3)	4,589 (54.9)	8,081 (32.2)
***Marital status***												
Single	101 (2.8)	646 (13.8)	1,689 (20.9)	4,499 (16.5)	34 (2.0)	643 (12.9)	2,116 (23.0)	4,791 (17.9)	39 (2.3)	663 (13.4)	2,120 (25.4)	4,657 (18.5)
Married	3,139 (86.3)	3,625 (77.5)	6,080 (75.1)	19,645 (72.2)	1,489 (86.9)	3,829 (76.6)	6,608 (71.9)	18,952 (70.8)	1,480 (85.5)	3,683 (74.2)	5,689 (68.1)	17,212 (68.5)
Others	399 (11.0)	406 (8.7)	332 (4.1)	3,057 (11.2)	190 (11.1)	524 (10.5)	467 (5.1)	3,036(11.3)	213 (12.3)	619 (12.5)	549 (6.6)	3,266 (13.0)
***Income* ^*b*^**	85.3 ± 83.7	127.4 ± 79.8	177.3 ± 110.3	132.2 ± 101.7	105.3 ± 77.2	163.6 ± 93.2	227.9 ± 132.0	180.1 ± 123.5	155.4 ± 140.5	210.2 ± 125.7	305.7 ± 176.1	234.8 ± 165.8
***Area***												
Rural	3,390 (93.2)	1,354 (29.0)	1,650 (20.4)	9,520 (35.0)	1,564 (91.3)	890 (17.8)	1,216 (13.2)	5,639 (21.1)	1,497 (86.4)	858 (17.3)	937 (11.2)	4,964 (19.7)
Urban	249 (6.8)	3,323 (71.1)	6,451 (79.6)	17,681 (65.0)	150 (8.8)	4,106 (82.2)	7,977 (86.8)	21,146 (79.0)	235 (13.6)	4,108 (82.7)	7,421 (88.8)	20,197 (80.3)
***Smoking status* ^*c*^**												
Never	681 (55.6)	598 (37.7)	1,343 (50.6)	4,965 (56.3)	237 (55.8)	572 (40.1)	1,531 (53.8)	4,762 (60.1)	264 (52.9)	579 (36.4)	1,414 (53.5)	4,354 (56.6)
Former	130 (10.6)	164 (10.3)	239 (9.0)	776 (8.8)	45 (10.6)	150 (10.5)	274 (9.6)	710 (9.0)	117 (23.5)	296 (18.6)	467 (17.7)	1,271 (16.5)
Current	414 (33.8)	825 (52.0)	1,075 (40.5)	3,082 (34.9)	143 (33.7)	706 (49.4)	1,038 (36.5)	2,449 (30.9)	118 (23.7)	716 (45.0)	763 (28.9)	2,069 (26.9)
***Alcohol consumption frequency* ^*c*^**												
None	560 (45.7)	371 (23.4)	630 (23.7)	2,952 (33.5)	236 (55.9)	546 (38.5)	1,152 (40.7)	3,975 (50.4)	279 (55.9)	566 (35.6)	1,031 (39.0)	3,718 (48.3)
≤ 2/week	334 (27.3)	717 (45.2)	1,310 (49.3)	3,875 (43.9)	106 (25.1)	589 (41.5)	1,291 (45.6)	2,923 (37.1)	98 (19.6)	644 (40.5)	1,214 (45.9)	2,829 (36.8)
≥ 3/week	331 (27.0)	499 (31.4)	717 (27.0)	1,996 (22.6)	80 (19.0)	285 (20.1)	391 (13.8)	982 (12.5)	122 (24.5)	381 (24.0)	399 (15.1)	1,147 (14.9)
***Medical check-up* ^*c *^ *(past 2 years)***												
Yes	533 (43.5)	859 (54.1)	1,599 (60.2)	4,268 (48.4)	195 (45.9)	754 (52.9)	1,581 (55.6)	3,799 (48.0)	282 (56.5)	848 (53.3)	1,520 (57.5)	3.906 (50.8)
No	692 (56.5)	728 (45.9)	1,058 (39.8)	4,555 (51.6)	230 (54.1)	671 (47.1)	1.262 (44.4)	4,116 (52.0)	217 (43.5)	742 (46.7)	1,124 (42.5)	3,787 (49.2)

Data were number *(%) *or mean ± SD

^a^Total population included farmers, manual workers, non-manual workers, and others

^b^Unit is 10 thousand Korean won/month

^c^Only a third of total participants had these information

The age-standardized prevalence of overall chronic disease and health service utilization by occupation according to the three calendar years are presented in Table 2. Female farmers reported significantly higher prevalence of doctor-diagnosed overall chronic diseases than other occupational groups and the total population, whereas male farmers showed a decreasing trend in prevalence of chronic diseases. Female farmers were less likely to report their general health as "good to excellent" than other groups. The numbers of doctor visits during the previous two weeks and reported hospitalizations were greater among female farmers than other groups in 2005.

**Table 2:** Age-standardized prevalence of chronic diseases and health service utilization by occupational groups and gender in three Korean National Health and Nutrition Examination Surveys

		1998		2001		2005	
	Cases	ASP (95%CI)^a^	Cases	ASP (95% CI)	Cases	ASP (95% CI)	Change (95% CI)^b^
Men							

***Overall chronic diseases* ^*c*^**							
Farmers	1,029	37.4 (34.1, 40.8)	533	35.2 (32.1, 38.5)	506	34.8 (29.6, 40.5)	-2.6 (-8.6, 3.6)
Manual	1,064	33.6 (31.4, 35.9)	1,209	34.4 (32.2, 36.7)	1,362	39.1 (37.1, 41.1)	5.5 (2.5, 8.5)
Non-manual	1,294	30.5 (28.5, 32.5)	1,532	33.3 (31.4, 35.2)	1,503	37.1 (35.3, 38.9)	6.6 (3.9, 9.3)
Total^d^	4,634	34.6 (33.3, 35.9)	4,585	35.4 (34.4, 36.5)	4,799	38.2 (37.2, 39.2)	3.6 (2.0, 5.3)

***Self-rated health "good to excellent"***							
Farmers	252	42.7 (35.7, 45.0)	89	62.3 (55.6, 68.5)	312	57.7 (51.4, 63.8)	15.0 (5.4, 24.7)
Manual	513	45.8 (42.4, 49.2)	498	50.3 (46.9, 53.7)	1,709	51.2 (49.1, 53.3)	5.4 (1.3, 9.5)
Non-manual	758	50.8 (47.3, 54.3)	855	55.2 (52.2, 58.1)	2,624	58.0 (56.0, 60.0)	7.2 (3.2, 11.2)
Total	1,916	46.6 (44.9, 48.3)	1,784	50.5 (48.7, 52.4)	5,825	52.7 (51.6, 53.9)	6.1 (4.0, 8.2)

***Doctor visits (past 2 weeks)***							
Farmers	592	25.4 (22.2, 28.8)	276	20.5 (15.9, 26.1)	326	29.5 (22.3, 37.8)	4.1 (-3.1, 11.3)
Manual	819	25.5 (23.5, 27.6)	573	16.3 (14.9, 17.9)	705	21.3 (19.7, 23.0)	-4.2 (-6.9, -1.6)
Non-manual	1,035	23.1 (21.3, 25.1)	639	15.1 (13.6, 16.7)	841	21.0 (19.4, 22.7)	-2.1 (-4.6, 0.3)
Total	3,212	24.7 (23.7, 25.6)	2,164	16.7 (16.0, 17.4)	2,723	21.5 (20.7, 22.4)	-3.2 (-4.4, -1.9)

***Hospitalizations (past 12 months)***							
Farmers	146	6.2 (4.7, 8.3)	50	7.0 (4.4, 11.1)	106	6.8 (5.2, 8.8)	0.6 (-2.2, 3.4)
Manual	150	4.7 (3.8, 5.7)	138	4.4 (3.4, 5.8)	307	9.8 (8.3, 11.5)	5.1 (3.3, 7.0)
Non-manual	162	3.6 (2.9, 4.5)	154	3.5 (2.7, 4.6)	261	6.1 (5.2, 7.1)	2.5 (1.2, 3.6)
Total	668	4.9 (4.5, 5.4)	539	4.2 (3.8, 4.5)	1,031	8.5 (7.9, 9.1)	3.6 (2.8, 4.2)

Women							

***Overall chronic diseases***							
Farmers	1,306	52.7 (49.6, 55.8)	610	57.4 (53.6, 61.0)	622	61.3 (50.1, 71.5)	8.6 (0.1, 17.2)
Manual	623	43.9 (40.8, 47.0)	669	48.9 (46.1, 51.8)	787	51.4 (48.1, 54.6)	7.5 (3.0, 12.0)
Non-manual	1,093	41.3 (39.0, 43.6)	1,312	43.5 (41.7, 45.2)	1,433	48.0 (46.3, 49.7)	6.7 (3.9, 9.6)
Total	6,934	46.9 (45.7, 48.2)	6,548	46.4 (45.5, 47.4)	7,029	50.4 (49.5, 51.4)	3.5 (1.9, 5.0)

***Self-rated health "good to excellent"***							
Farmers	200	37.9 (32.1, 44.0)	53	39.2 (29.3, 50.1)	144	28.7 (19.5, 40.2)	-9.2 (-22.2, 3.9)
Manual	190	41.8 (36.4, 47.5)	139	37.3 (31.7, 43.2)	481	35.1 (31.7, 38.7)	-6.7 (-13.7, 0.3)
Non-manual	514	45.5 (41.9, 49.1)	601	44.1 (41.0, 47.3)	1,993	45.8 (43.5, 48.1)	0.3 (-4.3, 5.0)
Total	1,801	38.4 (36.7, 40.0)	1,696	39.8 (38.0, 41.6)	5,158	40.3 (39.3, 41.3)	1.9 (-0.0, 3.9)

***Doctor visits (past 2 weeks)***							
Farmers	718	32.9 (29.1, 37.0)	299	23.6 (19.7, 27.9)	395	40.5 (33.2, 48.2)	7.6 (-0.1, 15.3)
Manual	485	33.4 (30.6, 36.3)	377	27.3 (24.4, 30.4)	453	28.7 (25.8, 31.9)	-4.7 (-8.8, -0.4)
Non-manual	1,015	35.7 (33.0, 38.5)	722	23.4 (20.5, 26.5)	920	30.2 (28.0, 32.5)	-5.5 (-9.0, -1.9)
Total	4,939	34.0 (32.9, 35.1)	3,486	24.6 (23.8, 25.4)	4,356	31.5 (30.6, 32.4)	-2.5 (-3.9, -1.1)
***Hospitalizations (past 12 months)***							
Farmers	96	6.7 (4.5, 10.0)	44	3.8 (2.3, 6.2)	83	16.2 (14.5, 18.0)	9.5 (5.2, 13.6)
Manual	41	3.3 (2.3, 4.6)	36	1.7 (1.2, 2.4)	112	7.0 (5.6, 8.8)	3.7 (1.7, 5.7)
Non-manual	202	5.3 (4.4, 6.5)	99	4.0 (2.4, 6.6)	258	8.4 (6.6, 10.6)	3.1 (0.8, 5.4)
Total	1,133	7.9 (7.4, 8.4)	493	3.5 (3.2, 3.9)	1,423	10.6 (10.0, 11.2)	2.7 (1.9, 3.5)

^a^Age-standardized prevalence and 95% confidence intervals

^b^Prevalence change from 1998 to 2005 and 95% confidence intervals

^c^Overall prevalence of chronic diseases was defined to be any chronic disease except dental diseases

^d^Total population included farmers, manual workers, non-manual workers, and others

Table 3 and 4 show selected chronic disease prevalence by occupational groups in men and women, respectively. The most prevalent diseases among farmers were arthritis, intervertebral disc disorders, and hypertension. Compared to other occupational groups and the total population, farmers had higher prevalence of arthritis and intervertebral disc disorders in both men and women, whereas male farmers had lower prevalence of hypertension and diabetes mellitus. Intervertebral disc disorders increased in all three occupational groups between 1998 and 2005, however, farmers had higher prevalence changes than other occupational groups during the study period.

**Table 3:** Age-standardized prevalence of selected chronic diseases among men by occupational groups in three Korean National Health and Nutrition Examination Surveys

		1998		2001		2005	
	Cases	ASP (95% CI)^a^	Cases	ASP (95% CI)	Cases	ASP (95% CI)	Change (95% CI)^b^
***Arthritis***							
Farmers	221	5.7 (4.9, 6.7)	117	6.1 (4.8, 7.8)	168	8.2 (6.8, 9.9)	2.5 (0.8, 4.1)
Manual	117	4.9 (3.9, 6.2)	101	3.6 (2.7, 4.9)	199	5.7 (4.9, 6.7)	0.8 (-0.7, 2.3)
Non-manual	69	1.8 (1.3, 2.3)	76	2.3 (1.6, 3.4)	119	4.1 (3.1, 5.5)	2.3 (1.2, 3.6)
Total^c^	588	4.1 (3.8, 4.6)	484	3.7 (3.3, 4.0)	790	5.7 (5.3, 6.2)	1.6 (0.5, 2.7)

***Intervertebral disc disorders***							
Farmers	78	3.3 (2.4, 4.6)	41	3.3 (2.1, 5.1)	87	8.6 (5.3, 13.8)	5.3 (2.0, 8.6)
Manual	72	2.0 (1.6, 2.6)	86	3.0 (2.1, 4.1)	184	5.0 (4.2, 5.9)	3.0 (2.0, 3.9)
Non-manual	88	2.0 (1.6, 2.5)	71	1.5 (1.0, 2.1)	147	3.9 (3.0, 5.0)	1.9 (0.8, 3.0)
Total	312	2.3 (2.0, 2.6)	286	2.2 (1.9, 2.5)	578	4.6 (4.2, 5.0)	2.3 (1.8, 2.8)

***Hypertension***							
Farmers	85	2.4 (1.9, 3.2)	94	4.5 (3.4, 5.9)	170	8.0 (6.5, 10.0)	5.6 (4.0, 7.1)
Manual	70	2.3 (1.6, 3.2)	248	6.6 (5.6, 7.8)	435	11.6 (10.5, 12.7)	9.3 (7.9, 10.6)
Non-manual	94	3.6 (3.1, 4.4)	263	7.0 (6.0, 8.2)	390	11.1 (9.8, 12.6)	7.5 (5.9, 8.9)
Total	384	2.8 (2.5, 3.2)	950	7.3 (6.8, 7.8)	1,589	11.7 (11.2, 12.3)	8.9 (8.2, 9.5)

***Chronic lung diseases***							
Farmers	72	2.3 (1.7, 3.3)	23	1.8 (1.0, 3.2)	22	1.6 (0.5, 4.7)	-0.7 (-2.4, 0.8)
Manual	50	1.5 (1.0, 2.2)	45	1.2 (0.8, 1.8)	47	1.2 (0.9, 1.7)	-0.3 (-1.0, 0.4)
Non-manual	56	1.4 (1.0, 1.9)	45	1.2 (0.8, 1.9)	40	1.4 (0.9, 2.2)	0.0 (-0.8, 0.8)
Total	262	1.9 (1.7, 2.2)	166	1.2 (1.0, 1.5)	184	1.4 (1.2, 1.6)	-0.5 (-0.9, -0.2)

***Asthma***							
Farmers	44	0.9 (0.6, 1.4)	29	1.2 (0.8, 1.8)	35	1.3 (0.9, 2.0)	0.4 (-0.2, 1.0)
Manual	20	1.2 (0.7, 2.2)	34	1.1 (0.7, 1.6)	46	1.4 (1.0, 2.0)	0.2 (-0.7, 1.1)
Non-manual	23	0.6 (0.4, 0.9)	31	1.0 (0.6, 1.9)	37	1.0 (0.6, 1.6)	0.4 (-0.1, 0.9)
Total	142	1.0 (0.8, 1.2)	162	1.3 (1.1, 1.5)	214	1.6 (1.4, 1.8)	0.6 (0.3, 0.9)

***Cancer***							
Farmers	14	0.3 (0.2, 0.6)	10	0.3 (0.2, 0.6)	15	0.5 (0.3, 0.9)	0.2 (-0.3, 0.5)
Manual	7	0.2 (0.1, 0.4)	10	0.3 (0.1, 0.6)	30	0.9 (0.6, 1.4)	0.7 (0.3, 1.1)
Non-manual	6	0.4 (0.1, 1.3)	12	0.4 (0.2, 0.8)	24	1.3 (0.7, 2.5)	0.9 (-0.1, 1.8)
Total	60	0.4 (0.3, 0.5)	87	0.7 (0.5, 0.8)	146	1.0 (0.9, 1.2)	0.6 (0.4, 0.8)

***Diabetes Mellitus***							
Farmers	39	1.2 (0.9, 1.7)	39	2.3 (1.5, 3.4)	71	3.8 (2.9, 5.1)	2.6 (1.7, 3.6)
Manual	45	1.6 (1.1, 2.3)	101	2.6 (2.1, 3.3)	180	4.7 (4.0, 5.6)	3.1 (2.2, 4.2)
Non-manual	57	1.6(1.1, 2.1)	146	4.0 (3.2, 5.0)	169	5.1 (4.1, 6.3)	3.5 (2.4, 4.7)
Total	234	1.8 (1.6, 2.1)	489	3.7 (3.4, 4.0)	728	5.4 (5.0, 5.8)	3.6 (3.1, 4.0)

***Cataract/glaucoma***							
Farmers	33	0.9 (0.6, 1.5)	16	0.7 (0.4, 1.1)	46	1.8 (1.3, 2.5)	0.9 (0.1, 1.6)
Manual	17	0.8 (0.4, 1.8)	19	0.8 (0.4, 1.5)	50	1.8 (1.2, 2.6)	1.0 (-0.0, 1.8)
Non-manual	20	0.9 (0.5, 1.7)	35	1.4 (0.9, 2.2)	39	1.4 (0.8, 2.5)	0.5 (-0.5, 1.5)
Total	120	0.9 (0.7, 1.1)	131	1.0 (0.8, 1.2)	296	2.6 (2.3, 2.8)	1.7 (1.4, 2.0)

^a^Age-standardized prevalence and 95% confidence intervals

^b^Prevalence change from 1998 to 2005 and 95% confidence intervals

^c^Total population included farmers, manual workers, non-manual workers, and others

**Table 4:** Age-standardized prevalence of selected chronic diseases among women by occupational groups in three Korean National Health and Nutrition Examination Surveys

		1998		2001		2005	
	Cases	ASP (95% CI)^a^	Cases	ASP (95% CI)	Cases	ASP (95% CI)	Change (95% CI)^b^
***Arthritis***							
Farmers	536	17.7 (15.8, 19.8)	301	18.9 (16.3, 21.9)	374	21.9 (18.9, 25.2)	4.2 (0.6, 7.8)
Manual	182	15.0 (12.8, 17.5)	189	14.7 (12.6, 17.1)	256	16.4 (14.4, 18.5)	1.4 (-1.7, 4.5)
Non-manual	213	10.2 (8.7, 11.9)	212	11.4 (9.0, 14.3)	267	17.2 (15.1, 19.5)	7.0 (4.2, 9.8)
Total^c^	2,084	13.6 (12.9, 14.3)	1,863	13.0 (12.4, 13.7)	2,536	17.2 (16.5, 17.8)	3.6 (2.6, 4.5)

***Intervertebral disc disorders***							
Farmers	106	4.1 (3.3, 5.1)	60	4.8 (3.5, 6.7)	125	9.9 (7.7, 12.5)	5.8 (3.6,7.8)
Manual	36	2.0 (1.4, 2.9)	48	3.7 (2.6, 5.3)	99	5.6 (4.4, 7.3)	3.6 (1.9, 5.3)
Non-manual	49	4.4 (3.7, 5.3)	88	3.3 (2.5, 4.2)	151	5.1 (3.8, 6.6)	0.7 (-1.0, 2.3)
Total	433	2.9 (2.7, 3.3)	450	3.1 (2.8, 3.4)	941	6.6 (6.1, 7.1)	3.7 (3.1, 4.2)

***Hypertension***							
Farmers	115	4.0 (2.9, 5.3)	104	5.8 (4.6, 7.4)	230	17.4 (11.8, 25.0)	13.4 (8.7, 18.4)
Manual	55	3.6 (2.5, 5.0)	109	8.1 (6.2, 10.4)	211	14.2 (12.1, 16.5)	10.6 (8.0, 13.2)
Non-manual	63	4.6 (3.2, 6.4)	142	7.1 (5.8,8.7)	231	14.5 (12.3, 17.0)	9.9 (7.0, 12.8)
Total	454	4.8 (4.4, 5.2)	877	9.3 (8.8, 10.0)	1,373	13.9 (13.4, 14.4)	9.1 (8.5, 9.8)

***Chronic lung diseases***							
Farmers	60	2.2 (1.4, 3.3)	16	0.9 (0.4, 2.2)	15	0.8 (0.5, 1.4)	-1.4 (-2.7, 0.0)
Manual	22	1.5 (0.9, 2.3)	16	1.0 (0.6, 1.6)	13	0.7 (0.4, 1.2)	-0.8 (-1.5, -0.1)
Non-manual	30	0.8 (0.5, 1.2)	34	1.0 (0.7, 1.6)	27	0.9 (0.6, 1.6)	0.1 (-0.5,0.7)
Total	259	1.7 (1.5, 2.0)	155	1.1 (0.9, 1.3)	138	0.9 (0.8, 1.1)	-0.8 (-1.1, -0.4)

***Asthma***							
Farmers	31	0.8 (0.5, 1.3)	18	1.0 (0.6, 1.5)	14	1.1 (0.6, 2.0)	0.3 (-0.5, 1.0)
Manual	13	0.8 (0.5, 1.4)	15	1.4 (0.6, 3.4)	35	2.1 (1.4, 3.2)	1.3 (0.3, 2.3)
Non-manual	27	0.9 (0.6, 1.6)	26	1.2 (0.7, 1.9)	37	1.5 (1.0,2.3)	0.6 (-0.3, 1.4)
Total	193	1.3 (1.1, 1.5)	198	1.4 (1.2, 1.7)	277	1.9 (1.7, 2.2)	0.6 (0.3, 1.0)

***Cancer***							
Farmers	7	0.2 (0.1, 0.5)	6	0.5 (0.2, 1.4)	5	0.3 (0.1, 0.8)	0.1 (-0.2, 0.4)
Manual	3	0.2 (0.1, 0.6)	6	0.3 (0.1, 0.8)	12	0.6 (0.3, 1.1)	0.4 (-0.01, 0.8)
Non-manual	8	0.7 (0.3, 1.7)	11	0.3 (0.2, 0.6)	21	0.8 (0.4, 1.5)	0.1 (-0.7, 0.9)
Total	59	0.4 (0.3, 0.6)	95	0.7 (0.6, 0.9)	142	1.0 (0.8, 1.2)	0.6 (0.3, 0.8)

***Diabetes Mellitus***							
Farmers	43	1.7 (0.9, 3.1)	41	2.9 (1.9, 4.5)	46	2.6 (1.8, 3.8)	0.9 (-0.7, 2.6)
Manual	13	1.3 (0.7, 2.4)	44	2.8 (2.0, 4.1)	53	2.9 (2.2, 3.8)	1.6 (0.5, 2.7)
Non-manual	25	1.1 (0.7, 1.7)	48	3.1 (2.2, 4.3)	65	5.1 (4.0, 6.6)	4.0 (2.6, 5.5)
Total	288	2.0 (1.8, 2.3)	486	3.4 (3.1, 3.8)	702	4.7 (4.4, 5.1)	2.7 (2.3, 3.2)

***Cataract/glaucoma***							
Farmers	44	1.6 (1.0, 2.4)	27	1.3 (0.9, 2.0)	64	3.6 (2.6, 5.0)	2.0 (0.8, 3.3)
Manual	11	1.0 (0.3, 3.0)	17	1.5 (0.8, 2.7)	54	4.4 (3.1, 6.3)	3.4 (1.5, 5.5)
Non-manual	14	1.2 (0.4, 3.2)	18	1.1 (0.6, 1.7)	36	3.9 (2.3, 6.7)	2.7 (0.3, 5.2)
Total	238	1.6 (1.4, 1.9)	242	1.7 (1.5, 2.0)	610	4.0 (3.7, 4.4)	2.4 (2.0, 2.8)

^a^Age-standardized prevalence and 95% confidence intervals

^b^Prevalence change from 1998 to 2005 and 95% confidence intervals

^c^Total population included farmers, manual workers, non-manual workers, and others

## Discussion

Using data from nationally representative samples of the South Korean population, we found that female farmers had a higher prevalence of overall chronic disease than other occupational groups, or the total population. Arthritis and intervertebral disc disorders were the most prevalent chronic diseases among farmers, and the increase in the prevalence of intervertebral disc disorders over time was greater among farmers than other groups.

The pronounced elevated prevalence of chronic diseases among women was consistent with previous findings. Female farmers from Finland have been reported to suffer more chronic diseases than female blue- and white-collar workers whereas male farmers' self-reported morbidity was on a par with that of other workers [14]. Female farmers were also more likely to poses higher rates of asthma, depression [15] and cancer of the lymphatic and hematopoietic system [2] than men in the US. Variances between male and female farmers indicate that lifestyle may be of different significance for the two genders. Women on farms may carry additional burdens associated with financial hardship, heavy seasonal workloads, exposure to chemicals, and experience employment off the farm, which may additionally contribute to the differences of disease prevalence between men and women [16,17].

High prevalence of arthritis and intervertebral disc disorders in farmers is consistent with previous studies that have indicated that farmers have higher rates of musculoskeletal diseases than other populations [18,19]. Numerous physical risk factors are integral to farming, such as high workloads, heavy lifting, bending and twisting, exposure to vibration from farm equipment, and performing tasks while in awkward postures [19]. These have been associated with an increased risk of acquiring musculoskeletal diseases such as osteoarthritis [20], low back pain [21,22], and neck and upper limb complaints [23]. The prevalence of musculoskeletal diseases and osteoarthritis among farmers were reported to be also higher than in other occupational groups in South Korea [24]. The increasing prevalence of arthritis and intervertebral disc disorders in farmers could also in part be explained by increased medical check-ups.

We found hypertension to be among the most prevalent diseases for farmers but the magnitudes of prevalence was lower than for other groups and the change of prevalence was also identified as the smallest among male farmers. These findings are consistent with other studies demonstrating that the prevalence of hypertension and associated risk factors are higher in urban than in rural areas [25,26]. In South Korea, the prevalence of hypertension for both genders was also reported to be higher in urban areas [27]. These prevalence differences between farmers and other groups may be related to the distribution of lifestyle factors [28]. In this study, farmers smoke less tobacco and drink less alcohol than other occupational groups (Table 1).

A slightly higher prevalence of chronic lung diseases was seen in male farmers than in other occupational groups and the total population. Previous studies have shown that farmers are exposed to a variety of inhaled agents including inorganic/organic dusts, microorganisms, mycotoxins, endotoxins, pollens, mites, molds, animal production, and pesticides [29]. These exposures have been demonstrated to give rise to a variety of respiratory disorders [30,31]. In South Korea, it was reported that the level of organic dusts and ammonia exceeded in poultry farms [32] and that respiratory diseases were reported frequently among farmers [11]. Since the proportion of smokers among farmers was lower than within other groups in this study, the contribution of farming for this difference in prevalence would be significant.

Farmers showed a slightly lower prevalence of total cancer than other groups in 2005. Previous studies also pointed out a remarkable deficit in total cancer compared with the total population in the United States [3,4], Sweden [33], Finland [34] and Italy [35]. Several factors may contribute to the lower rate of total cancer including a healthier lifestyle manifested by lower cigarette use and an occupation that has traditionally required high levels of physical activity. However, previous ecologic study in South Korea demonstrated that rural areas retain an increased risk of cancer mortality [36] over the total population. This discrepancy between prevalence and mortality may in part be explained by rural population general entrance into the health care system at a later point and with more advanced stages of diseases than urban residents due to lower incomes and less education [37].

Our study found a lower prevalence of diabetes mellitus among male farmers than among others, consistent with other studies. Mortality from diabetes showed a decreased risk in farming [4], whereas prevalence data are mixed [26]. Healthy diet as a part of rural lifestyles may explain these findings. However, the Agricultural Health Study reported that exposure to certain pesticides increased the risk of diabetes [38]. Due to its high occurrence and the equivocal findings, diabetes mellitus should be further investigated among farmers.

There are important limitations of this study. First is the cross-sectional nature of the data, which may lead to an underestimation of the true health situation, since sick people may be absent from the work force. However, this does not pose a major limitation to investigating changes and to comparison with other groups because data from the three consecutive surveys were used which apply identical disease criteria. Second is the reliance on self-reported medical history potentially leading to misclassification. Self-reported data, however, are reasonably assumed to be accurate for selected chronic diseases and are commonly used to estimate the prevalence of health conditions [39,40]. We also included only doctor-diagnosed diseases to minimize this issue in the study. However, we assume that underestimation of some chronic diseases such as hypertension and diabetes mellitus undoubtedly occurs. Finally, we were unable to examine separate prevalence by detailed occupational groups (i.e., farmers, fishery, and forestry workers) because of limitations in the information collected by the KNHNES. Fishery and forestry workers were reported to have higher rates than the total population of fatal accidents, injury, musculoskeletal diseases, and respiratory diseases [41]. However, the population of fishery and forestry workers made up less than 5% of the farmer group in our study. Therefore, the estimates of prevalence and changes would be mainly explained by farmers.

The strength of this study includes the nationally representative nature of the large sample sizes, which are superior datasets for estimating prevalence. In addition, the use of other occupational workers and the total population as reference groups has an advantage in comparability of disease prevalence.

## Conclusion

We report that South Korean farmers displayed a higher prevalence of doctor-diagnosed arthritis and intervertebral disc disorders than other occupational groups and the total population, and that the trend of those diseases is increasing with time. This study has provided the additional information that farmers have a distinct pattern of disease prevalence from other populations and supports that the primary focus in agricultural health in South Korea should be on addressing this pattern. Therefore, more detailed studies investigating the risk of these diseases and intensive intervention efforts to reduce the prevalence of musculoskeletal diseases, particularly among women, are required for South Korean farmers.

## List of abbreviations

KNHNES: Korean National Health and Nutrition Examination Survey.

## Competing interests

The authors declare that they have no competing interests.

## Authors' contributions

ESC performed the statistical analysis and drafted the manuscript. KAK provided substantive statistical advice and helped interpret the data. EKM carried out the acquisition of data and helped interpret the results. WJL conceived of the study, and participated in its design and coordination and helped to draft the manuscript. All authors revised drafts of the manuscript and read and approved the final manuscript.

## Pre-publication history

The pre-publication history for this paper can be accessed here:

http://www.biomedcentral.com/1471-2458/9/268/prepub
